# Pathology-Driven Automation to Improve Updating Documented Follow-Up Recommendations in the Electronic Health Record After Colonoscopy

**DOI:** 10.14309/ctg.0000000000000785

**Published:** 2024-11-13

**Authors:** Elizabeth R. Stevens, Arielle Nagler, Casey Monina, JaeEun Kwon, Amanda Olesen Wickline, Gary Kalkut, David Ranson, Seth A. Gross, Aasma Shaukat, Adam Szerencsy

**Affiliations:** 1 Department of Population Health, NYU Grossman School of Medicine, New York, New York, USA;; 2 Department of Health Informatics, NYU Langone Health, New York, New York, USA;; 3 Department of Dermatology, NYU Langone Health, New York, New York, USA;; 4 Medical Center Information Technology, NYU Langone Health, New York, New York, USA;; 5 Department of Medicine, New York University Grossman School of Medicine, New York, New York, USA;; 6 Division of Gastroenterology and Hepatology, NYU Langone Health, New York, New York, USA.

**Keywords:** colonoscopy, colorectal cancer screening, clinical decision support, rules engine, guideline concordance, automation

## Abstract

**INTRODUCTION:**

Failure to document colonoscopy follow-up needs postpolypectomy can lead to delayed detection of colorectal cancer (CRC). Automating the update of a unified follow-up date in the electronic health record (EHR) may increase the number of patients with guideline-concordant CRC follow-up screening.

**METHODS:**

Prospective pre-post design study of an automated rules engine-based tool using colonoscopy pathology results to automate updates to documented CRC screening due dates was performed as an operational initiative, deployed enterprise-wide May 2023. Participants were aged 45–75 years who received a colonoscopy November 2022 to November 2023. Primary outcome measure is rate of updates to screening due dates and proportion with recommended follow-up < 10 years. Multivariable log-binomial regression was performed (relative risk, 95% confidence intervals).

**RESULTS:**

Study population included 9,824 standard care and 19,340 intervention patients. Patients had a mean age of 58.6 ± 8.6 years and were 53.4% female, 69.6% non-Hispanic White, 13.5% non-Hispanic Black, 6.5% Asian, and 4.6% Hispanic. Postintervention, 46.7% of follow-up recommendations were updated by the rules engine. The proportion of patients with a 10-year default follow-up frequency significantly decreased (88.7%–42.8%, *P* < 0.001). The mean follow-up frequency decreased by 1.9 years (9.3–7.4 years, *P* < 0.001). Overall likelihood of an updated follow-up date significantly increased (relative risk 5.62, 95% confidence intervals: 5.30–5.95, *P* < 0.001).

**DISCUSSION:**

An automated rules engine-based tool has the potential to increase the accuracy of colonoscopy follow-up dates recorded in patient EHR. The results emphasize the opportunity for more automated and integrated solutions for updating and maintaining EHR health maintenance activities.

## Graphical abstract

**Figure:**
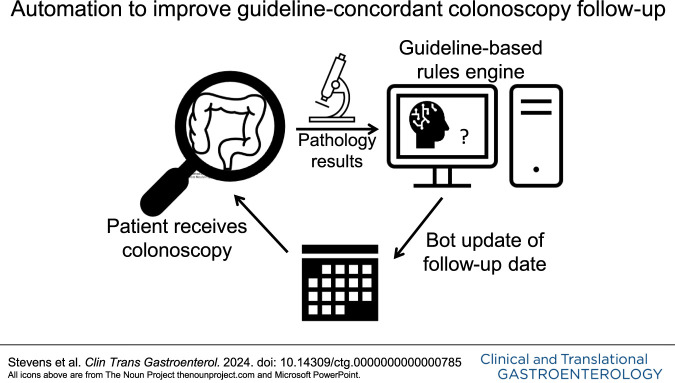

## INTRODUCTION

Colorectal cancer (CRC) is the second most common cause of cancer deaths in the United States (1). CRC screening is a critical aspect of preventive health care, having been associated with a significant decrease in CRC incidence and mortality rates (2). Furthermore, findings on colonoscopy can be used to determine the future risk of CRC and the need for more frequent surveillance. Failure to document the appropriate colonoscopy follow-up, however, can lead to missed colonoscopies and delayed detection of CRC.

The responsibility of documenting the timing for follow-up screening frequently falls to the provider; however, keeping track of a patient's next recommended due date for screening can be administratively burdensome for clinicians, especially when dealing with large volumes of patients with complex needs and varying potential colonoscopy findings (3). Although many electronic health records (EHRs) have automated reminders to assist in managing CRC screening follow-up, the integration of systems to track abnormalities and manage follow-up is limited. For many preventative care activities, EHR systems do not automatically integrate data from other areas of the EHR, leaving documentation of care plans that deviate from standard recommendations to be performed manually, placing a high burden on clinicians, and leading to substantial missed opportunities for improved preventative care (4,5).

EHR tools such as the Epic Systems Health Maintenance (HM) activity, use dates of prior completed screening and data manually entered by providers to track screening events, and create alerts for upcoming screening due dates. However, while the HM activity automatically captures the completion of CRC screening, documenting the follow-up date fails to incorporate pathology findings or patient-specific factors. Instead, the EHR defaults to a standard screening follow-up date of tentatively due in 10 years, as per the US Multi-Society Task Force (USMSTF) on CRC guidelines for normal-risk adults (4). Therefore, each patient with a significant finding on colonoscopy requires a manual update of the HM activity to recall a patient sooner for follow-up (6). With polyps found during colonoscopy in over 30% of people aged older than 45 years (7), this amounts to a sizeable population of patients who are likely to require a more frequent colonoscopy.

Automating the update of the EHR screening reminder for abnormal colonoscopy results is likely to increase the number of patients with documented guideline-concordant CRC follow-up screening interval (8). As part of an operational quality improvement initiative, we developed an innovative rules-based clinical decision support (CDS) tool to automate updates to the patient's HM follow-up date for CRC screening based on pathology results with the objective of improving the rate of documented CRC follow-up interval in concordance with guideline recommendations while reducing provider documentation burden. We then conducted a pre-post analysis to measure the impact of the CDS tool implementation on the HM CRC follow-up recommendations.

## METHODS

This organization-wide pre-post study sought to measure the effect of implementing an automated rules engine-based CDS tool designed to update the recommended CRC follow-up dates based on pathology results from polypectomy. As an operational quality improvement project, the NYU Langone Health Institutional Review Board waived approval for the study procedures. Within the study procedures, a waiver of Health Insurance Portability and Accountability Act (HIPAA) authorization was included to access patient medical records. All study procedures complied with institutional ethical standards and the standards set by the Declaration of Helsinki.

### Study setting and participants

EHR data were extracted for patients with a colonoscopy completed between November 2022 and November 2023. The NYU Langone Health (NYULH) system is a private hospital system serving the greater New York area and Florida with approximately 6.8 million active patients. The NYULH system uses Epic Systems software for the management of its EHR. To be included in the study, a patient must: (i) have a colonoscopy completed between November 2022 and November 2023 and (ii) be at the age of 45–75 years (starting at 40 years for those with a family history of CRC). The same individual patients were not specifically observed in both the preintervention and postintervention phases of the study. Patients with a documented HM follow-up date of less than 1 year were excluded from the study as they do not fall within USMSTF guidelines and are likely indicative of the need for a repeat colonoscopy due to a cancer diagnosis, low quality bowel preparation, or human error (e.g., a typo).

### Study design

This study was an organization-wide prospective pre-post design that compared standard care with the effect of an automated, EHR-based rules engine CDS tool on the frequency of the follow-up documented in the patient's EHR (i.e., HM section).

#### Standard care

In standard care, a patient's list of preventive care items is maintained separate from clinical notes in the EHR's HM section. The HM is used to remind clinicians and staff about completed and upcoming preventive health tests and procedures, including CRC screening. In accordance with the USMSTF CRC guidelines (9), NYULH configured the HM activity to begin screening for colon cancer at the age of 45–75 years for average-risk patients (starting at 40 years for those with a family history of CRC) and default to a 10-year follow-up screening. The HM activity is not directly linked to clinical notes or test results otherwise present in the EHR, and the HM section must be manually modified to reflect any non-10-year follow-up screening frequency recommendations based on pathology findings or patient-specific conditions. Patients receiving a colonoscopy between November 2022 and the intervention implementation (May 10, 2023) were considered standard care and served as the study control group.

#### Intervention

The intervention was designed to automate updating the default HM follow-up date, when appropriate, based on pathology results from polypectomy following colonoscopy. Importantly, clinicians remain the final decision makers and remain able to customize the next follow-up date based on patient-specific factors. The intervention is a rules engine CDS tool based on components of the USMSTF guidelines for follow-up after colonoscopy and polypectomy (10). The CDS tool uses a ruleset (Table 1) to update a patient's next colon cancer screening date in the HM activity. Instead of defaulting the screening interval to a standard 10-year follow-up, the new rules engine modifies a patient's screening interval to 3, 5, 7, or 10 years based on the pathology results after polypectomy according to clinical guidelines. When the USMSTF guidelines provided a range for screening interval, the shorter follow-up date time was selected. Pathology findings that would result in a follow-up frequency less than 3 years were not automated.

**Table 1.** Screening frequency rules by pathology finding

	Screening frequency		
	3 yr	5 yr	7 yr
Pathology finding	Tubulovillous adenoma	Sessile serrated polyp/adenoma	Tubular adenoma, 1-2
	Tubulovillous adenoma, multiple fragments	Sessile serrated polyp(s)/adenoma(s) w/multiple fragments	Tubular adenoma(s), multiple fragments
	Tubulovillous adenoma w/high-grade dysplasia	Sessile serrated lesion	Tubular adenoma(s), fragments
	Tubulovillous adenoma, fragments		
	Traditional serrated adenoma		
	Tubular adenoma w/high-grade dysplasia		
	3 or more Dx only: Tubular adenoma		
	3 or more Dx only: Tubular adenoma(s), multiple fragments		
	3 or more Dx only: Tubular adenoma(s), fragments		

The intervention decision rules were developed with an integrated group of clinician subject matter experts at NYULH based on USMSTF guidelines for follow-up after colonoscopy and polypectomy (10,11). This group consisted of gastroenterologists, pathologists, physician informaticists along with health system clinical leadership and clinical systems analysts. Of note, the rules were unable to incorporate polyp size, as the size at the time of pathological evaluation does not match the gross size used to create screening interval guidelines. To configure the rules engine, all GI-related pathology findings needed to be discretely coded within the EHR-based pathology application (Epic Beaker). As opposed to prerules engine usual practice of using free text, once coded, pathologists prospectively selected relevant diagnoses from a discrete list (Table 1), taking into consideration the polyp size and number of specimens to account for the other components of the recommendations. The combination of these findings was programmed into a rules engine in the pathology application which automatically adjusted the HM activity follow-up date accordingly. In addition to updating the HM section, the next follow-up date is displayed in the pathology result report in the EHR to provide greater visibility. The intervention was implemented enterprise-wide May 10, 2023.

#### Intervention validation

A retrospective chart review was performed to evaluate the rate of concordance between the rules engine HM decision, the USMSTF guidelines for follow-up after colonoscopy and polypectomy, and provider recommendation (10,11). A simple random sample of 100 patients was selected for the chart review. Two authors (E.R.S., A.S.) reviewed the colonoscopy report, pathology results, and any provider notes to determine guideline appropriate follow-up screening interval and assess any patient-specific factors that may have influenced provider recommendations (e.g. high risk, bowel prep, etc). These findings were compared with the rules engine decision and categorized as concordant or discordant with USMSTF guidelines, and secondarily concordant or discordant with the provider recommendation.

### Measures

The primary outcome measure assessed includes the number of updates made to the HM activity based on pathology results and proportion of HM activity recommended follow-up less than the 10-year default (10). HM activity updates were categorized into changes made by the rules engine and manually by clinicians. When changes were made by both the rules engine and the clinician, the patient was labeled as being clinician updated. Additional patient demographic characteristics measures were collected including age, sex, and race/ethnicity.

### Statistical analysis

We conducted descriptive statistics to summarize patient demographics in the preintervention and postintervention periods. Rao-Scott χ^2^ tests (for categorical variables) and ANOVA (for continuous variables) compared the difference between pre-intervention and postintervention. Multivariable log-binomial regression was performed to examine the associations between the intervention and HM update to <10 years, as well as differences in update rates by patient demographics. The results for regression models were recorded as a relative risk (RR) with 95% confidence intervals (95% CIs). The level of statistical significance was set at *P* < 0.05. To address potential confounding by the updates to the HM by clinicians, we used the Cochran-Mantel-Haenszel Method (12) (M-H) to calculate an M-H RR of updates stratified by clinician update status. All data analyses were performed using R (13).

## RESULTS

**Figure 1.**
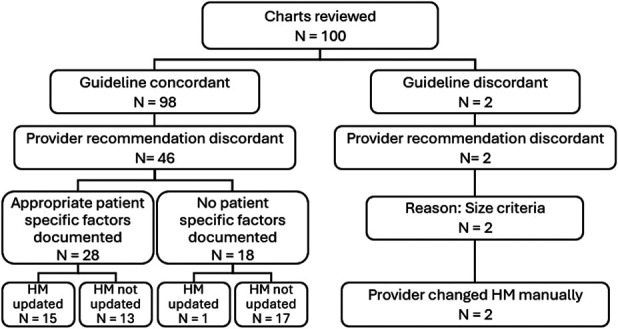
Rules engine validation chart review outcomes. HM, health maintenance.

Ninety-eight percent (98) of the 100 rules engine HM decisions reviewed were deemed guideline concordant (Figure 1). Of the guideline-concordant recommendations, 46.9% (46) had a provider recommendation for follow-up shorter than the guideline recommendation. Of those patients, 54.3% (25) were at high risk due to personal or family history, 6.5% (3) had poor bowel preparation, and 15.2% (7) had no identified factor for a shortened surveillance period. The provider manually changed the HM follow-up for 32.6% (15) of these patients. Of the 100 patient charts reviewed for validation, 2% (2) were identified as having a guideline-discordant rules engine HM decision. Of those discordant decisions, both were due to patients with polyps > 10 mm, which is not accounted for in the rules engine, and both were manually updated by the provider.

The study population consisted of 29,164 patients including 9,824 receiving usual care and 19,340 receiving the intervention, 22 patients were removed due to a follow-up of <1 year. Table 2 presents the distribution of demographic and HM characteristics of the overall study population and by study arm. The study population had a mean age of 58.6 ± 8.6 years and included an estimated 53.4% female patients, 69.6% non-Hispanic White, 13.5% non-Hispanic Black, 6.5% Asian, and 4.5% Hispanic patients. Small, but statistically significant differences were observed between the preintervention and postintervention groups in mean age (58.2 vs 58.7 years, *P* < 0.001), Hispanic race (3.9% vs 4.8%, *P* < 0.001), and female (52.5% vs 53.8%, *P* = 0.04).

**Table 2.** Participant and HM activity characteristics by the intervention group

	Overall % (n)	Preintervention % (n)	Postintervention % (n)	*P* value
	n = 29,164	n = 9,824	n = 19,340	
Age (mean [SD])	58.6 (8.6)	58.2 (8.6)	58.8 (8.7)	<0.001
Female	53.4 (15,570)	52.5 (5,161)	53.8 (10,409)	0.04
Race				
Asian	6.5 (1,687)	6.4 (559)	6.5 (1,128)	0.72
Black, non-Hispanic	13.5 (3,518)	13.7 (1,199)	13.4 (2,319)	0.49
Hispanic	4.5 (1,148)	3.9 (339)	4.8 (831)	<0.001
White, non-Hispanic	69.6 (18,090)	70.3 (6,131)	69.3 (11,959)	0.09
Other, non-Hispanic	4.6 (1,204)	4.7 (409)	4.6 (795)	0.78
Clinician updated	11.1 (2,	11.5 (1,	11.0 (2,	0.20
Rules engine updated	—	—	46.7 (9,	—
HM follow-up, yr				
10	58.2 (16,987)	88.7 (8,712)	42.8 (8,275)	<0.001
7	19.4 (5,658)	0.3 (34)	29.1 (5,624)	
5	11.4 (3,312)	6.0 (592)	14.1 (2,720)	
3	9.5 (2,764)	3.5 (343)	12.5 (2,421)	

HM, health maintenance activity.

**Figure 2.**
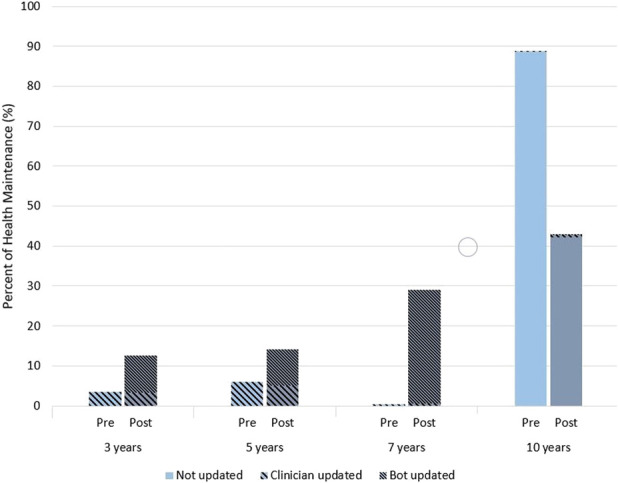
Health maintenance activity follow-up frequency before and after intervention implementation.

Postintervention, 46.7% of HM activity follow-up recommendations were updated by the rules engine and 11.0% were changed by a clinician (Table 2). One thousand four hundred thirty-nine patients had both a clinician and rules engine update recorded in their HM activity. Postintervention, the proportion of patients with HM activities with a 10-year default follow-up frequency significantly decreased (88.7%–42.7%, *P* < 0.001). HM activity follow-up recommendation for 7-year, 5-year, and 3-year follow-ups significantly increased postintervention (0.3%–29.1%, 6.0%–14.1%, and 3.5%–12.5%, respectively, *P* < 0.001) (Figure 2).

The intervention was associated with a more than 5 times chance that a patient would have a HM colonoscopy screening frequency of less than 10 years (RR 5.62, 95%CI: 5.30–5.95, *P* < 0.001). There were no significant associations between patient demographic characteristics and a HM colonoscopy screening frequency of less than 10 years.

## DISCUSSION

In this study, we found that the use of a rules engine-based CDS tool designed to automatically update the recommended colonoscopy follow-up date based on pathology results had high validity and increased the likelihood of our patients' HM next follow-up date being updated by more than 5-fold, while still enabling clinicians to manually set or recall patients sooner for their next colonoscopy if needed. After intervention implementation, most patients received a recommended screening follow-up date of less than the standard default of 10 years. This indicates the potential for automated CDS tools to provide a more consistent and accurate recommended colonoscopy follow-up date, as compared with relying on clinician action alone.

These findings are consistent with previous research demonstrating that the use of CDS tools can increase the likelihood of guideline-concordant preventative care activities (14–16). Using mechanisms similar to those of other CDS tools—by integrating clinical guidelines into the process of determining colonoscopy screening follow-up frequency—our CDS tool significantly increased the likelihood of an update to the HM colonoscopy follow-up date. Moreover, our validation study identified a notable portion of providers that requested a follow-up period shorter than USMSTF guideline recommendations. Therefore, the introduction of the HM automation may increase the guideline-concordant care to a greater extent than having all HM updated manually by providers. The rules engine-based CDS tool examined in this study is novel in its use of automation, triggered by pathology results, to update the HM activity without clinician intervention.

By eliminating the need for a clinician to initiate an update to the HM activity, this tool both increases the likelihood of updated guideline-concordant screening frequency documentation and decreases clinician documentation burden. Thus, achieving the US Safety Assurance Factors for EHR Resilience Guides goals (17) and their supplementary SMARTER Guide's aim to improve EHR cognitive support by automating routine tasks and automatically translating actions into documentation (5). This means of automating an update of the HM activity based on test results is likely to be applicable to other areas of care, and the success of this tool can likely be extended to other guideline-based HM activities. Indeed, based on the results of this operational study, our institution has elected to retain the use of this CRC CDS tool and is currently investigating additional application areas.

Disparate areas for documenting screening follow-up recommendations within EHR can result in discrepancies within the HM activity, demanding careful and manual—sometimes even duplicative—review and adjustments, which are a resource and time-intensive process in an already burdened setting. Discrepancies in the report and the system can propagate misinformation, causing confusion about when patients should return for repeat screening and surveillance. The rules engine-based CDS tool in this study represents an effective solution to reduce discrepancies in most patient EHR HM activities without increasing burden on clinicians to manually update patient records. However, in our study, a small number of patient follow-up dates were updated by both the CDS tool and a clinician. These duplicative updates did not have a prevailing pattern, with some clinicians shortening the HM follow-up, others extending it, and still others just manually verifying the update already made by the rules engine. Therefore, future research is needed to determine the impact of the automatic update on guideline concordance and on clinical outcomes, including the receipt of guideline-concordant colonoscopy follow-up and CRC detection.

The rules engine tool in this study, however, is limited in its ability to update the HM activity to account for all recommendations in the USMSTF on CRC guidelines and risk factors independent of pathology findings in high-risk patients with more complex diagnoses such as cancers. Therefore, further research is needed to develop additional tools with the capability to draw on more than the EHR pathology results. The inclusion of technologies such as natural language processing and generative artificial intelligence may hold a solution to extracting relevant patient data for use in CDS algorithms from EHR data that can often be unstructured and inconsistent in nature (e.g. clinician notes). The use of integrated tools such as Lumens Epic (18), that provide readily accessible endoscopy data within the EHR system, may also reduce the burden of data coding, allowing for increased specificity—including adenoma size—for codes used. Similarly, further advancements in this type of rules engine would likely benefit from the ability to incorporate external patient data from point-to-point record exchange tools such as Epic Systems Corporation's Care Everywhere (19). The inclusion of these addition/alternate sources of patient data is likely to ensure greater HM activity accuracy, particularly for those with complex risk circumstances.

### Limitations

This study had several limitations. First, due to the enterprise-wide roll out of the program, the study had no designated control group. Therefore, preintervention and postintervention update rate changes resulting from population or temporal changes cannot be entirely ruled out. Second, the CDS algorithm does not consider polyp size, family history, or other patient-specific risk factors outside of the pathology results. Therefore, the changes made by the rules engine may not represent complete guideline concordance among those with additional risk factors. Manual validation in a sample of results, however, indicates a high validity of the CDS and strong concordance of the rules engine decisions with guideline recommendations for pathology findings despite adenoma size. However, as the rules engine still allows for manual update of the HM activity, the rate of inappropriately long follow-up recommendations is anticipated to be no greater than, and likely much lower than, that of usual care. Similarly, when the USMSTF on CRC guidelines provided a range for suggested follow-up, the rules engine was programmed to select the shorter follow-up time. This decision was made to provide a more conservative follow-up time frame. However, there is clinical equipoise on whether shorter surveillance is better than longer surveillance for patients with low-risk adenomas. Therefore, this decision on how to use recommendation ranges should be reassessed when more evidence becomes available, such as from the ongoing European Polyp Surveillance (20) and the 5-year or 10-year colonoscopy for 1–2 nonadvanced adenomatous polyps trials (21). Third, the intervention does not apply to colonoscopies outside our Epic EHR. Finally, the HM activity field used in this study was specific to the Epic EHR and may not be applicable to other EHR systems.

Failure to update follow-up dates for recommended screening can lead to missed colonoscopies and delayed detection of CRC. The implementation of an automated rules engine-based CDS tool using colonoscopy pathology results has the potential to increase the accuracy of colonoscopy follow-up screening dates recorded in patient EHR HM activities without increasing clinician documentation burden. The results of this study emphasize the need for more automated and integrated solutions for updating and maintaining EHR HM activities.

## CONFLICTS OF INTEREST

**Guarantor of the article:** Elizabeth R. Stevens, PhD, MPH.

**Specific author contributions:** A.N., S.A.G., A.S., A.Sz.: study conception. E.R.S., A.N., C.M., A.O.W., G.K., D.R., S.A.G., A.S., A.Sz.: study methods. C.M., A.O.W., D.R.: data curation. E.R.S., A.N., C.M., A.S.: data analysis and interpretation. E.R.S., J.K., A.S.: original manuscript draft. E.R.S., A.N., C.M., J.K., A.S., A.Sz.: manuscript revision. All authors approved the final submitted draft.

**Financial support:** None to report.

**Potential competing interests:** None to report.

**Study Highlights**

**WHAT IS KNOWN**

✓ Failure to document colonoscopy follow-up needs delays in colorectal cancer detection.
✓ Documentation of colonoscopy follow-up needs often falls onto the provider.
✓ Many patient's colonoscopy follow-up needs are not accurately documented.

**WHAT IS NEW HERE**

✓ An automated rules engine increased the accuracy of recorded colonoscopy follow-up dates.
✓ Automation increased likelihood of follow-up date being updated by more than 5-fold.
✓ After intervention, most patients had a screening follow-up date less than the standard of 10 years.
